# Cardiometabolic Mortality and Health System Expansion in Kuwait (2010–2022): A National Time-Series Analysis

**DOI:** 10.3390/jcm15072697

**Published:** 2026-04-02

**Authors:** Ahmad Salman

**Affiliations:** Department of Public Health Practice, College of Public Health, Kuwait University, Sabah Al-Salem University City, Al-Shadadiya, Safat, Kuwait City 13110, Kuwait; ahmad.salman@ku.edu.kw

**Keywords:** cardiometabolic mortality, cardiovascular disease, ischaemic heart disease, diabetes mellitus, non-communicable diseases, modifiable risk factors, disease prevention, health system expansion, Gulf Cooperation Council, Kuwait, time-series analysis, ecological study

## Abstract

**Background**: Cardiometabolic diseases are a leading cause of premature mortality globally, yet longitudinal national mortality patterns remain insufficiently characterised in Gulf Cooperation Council settings. This study examines national trends in cardiometabolic mortality alongside health system financing, capacity, and utilization in Kuwait between 2010 and 2022. **Methods**: A national ecological time-series analysis used Ministry of Health administrative data covering mortality, cardiac care unit (CCU) capacity and discharges, cardiovascular procedural volumes, and MOH expenditure. Cause-specific outcomes included circulatory disease, ischaemic heart disease (IHD), cerebrovascular disease, hypertensive disease, and diabetes mellitus. Ordinary least squares regression estimated annual trends; pre-COVID restricted models (2010–2019) separated secular from pandemic-period effects. **Results**: All-cause deaths rose significantly from 5448 (2010) to 8041 (2022; β = +373.5/year; *p* = 0.001), peaking at 10,938 in 2021. Circulatory disease mortality rates increased over the full series but not pre-COVID, indicating pandemic-era acceleration. IHD death counts rose significantly in both models (β = +68.4 and +67.0/year; *p* < 0.01); IHD rates showed no significant trend, implicating demographic growth. Diabetes demonstrated the strongest signal: significant increases in death counts (β = +36.5/year; *p* < 0.001) and mortality rates (β = +0.689/100,000/year; *p* = 0.002), rising progressively across all time blocks. Hypertensive mortality declined significantly (β = −0.113/year; *p* = 0.002). MOH expenditure, CCU capacity, and CCU discharges increased significantly, demonstrating sustained structural expansion of cardiovascular services. **Conclusions**: Rising cardiometabolic mortality—driven prominently by diabetes—occurred alongside sustained health system expansion in Kuwait, indicating that tertiary capacity growth alone is insufficient to offset underlying epidemiological pressures. These findings underscore the urgency of strengthening upstream cardiometabolic prevention, integrated diabetes surveillance, and long-term metabolic risk control as central pillars of sustainable NCD policy.

## 1. Introduction

Non-communicable diseases (NCDs) account for approximately 74% of global deaths, with cardiovascular diseases (CVDs) representing the leading cause worldwide, followed by cancer, chronic respiratory diseases, and diabetes mellitus [1]. In 2019, CVD alone accounted for an estimated 18.6 million deaths globally, reflecting both persistent metabolic risk factor burdens and demographic shifts including population growth and ageing [2,3]. While age-standardised cardiovascular mortality has declined in many high-income countries over recent decades, absolute death counts remain substantial and progress has plateaued across several regions [2,3]. Together, these trends reflect the persistent and interconnected burden of cardiometabolic diseases at the population level.

Diabetes mellitus represents a closely linked and rapidly expanding component of this burden. The International Diabetes Federation (IDF) estimated that 537 million adults were living with diabetes in 2021, with projections rising to 643 million by 2030 [4]. Diabetes contributes directly to mortality and indirectly through cardiovascular complications, reinforcing the integrated concept of cardiometabolic disease [4,5]. Global Burden of Disease (GBD) analyses have demonstrated that diabetes-related mortality and metabolic risk exposure have increased across multiple regions despite improvements in acute cardiovascular care [2,6].

The Gulf region, including Kuwait, is characterised by high prevalence of obesity, physical inactivity, and type 2 diabetes [7,8]. Kuwait ranks among the countries with the highest diabetes prevalence globally, with adult prevalence estimates exceeding 15–20% in recent national and regional reports [4,8]. Rapid urbanisation, sedentary lifestyles, and dietary transitions have contributed to sustained cardiometabolic risk across the population [7,9]. GCC countries report a substantial NCD-related mortality burden, with CVD representing the leading cause of NCD death across member states [10,11].

Against this epidemiological backdrop, Kuwait has substantially expanded its health system over the past decade, consistent with broader trends across GCC states [10,11]. Ministry of Health (MOH) expenditure, tertiary care capacity, and cardiovascular service infrastructure have grown markedly, including increases in coronary care unit (CCU) beds, interventional cardiology services, and specialist outpatient provision. However, expansion of health system inputs does not necessarily translate into reductions in population-level mortality, particularly when upstream metabolic risk factors remain uncontrolled. Evidence from large-scale prospective cohort research demonstrates that the majority of CVD events and deaths are attributable to modifiable upstream risk factors—including hypertension, diet, physical inactivity, and metabolic dysregulation—which clinical care alone cannot adequately address [12]. Upstream prevention, metabolic risk control, and continuity of chronic disease management therefore remain central determinants of long-term cardiometabolic outcomes alongside advances in acute care.

The COVID-19 pandemic introduced additional complexity into mortality patterns worldwide. Excess mortality has been documented across multiple countries, reflecting both direct viral effects and indirect disruptions to chronic disease management and healthcare utilisation [13,14]. Studies from the United States and Europe have reported temporary increases in cardiovascular mortality and interruptions in elective cardiac procedures during the pandemic period, with similar patterns observed across multiple health systems [15,16]. Distinguishing long-term secular mortality trends from pandemic-associated deviations is therefore essential for accurate interpretation of recent cardiometabolic mortality patterns.

Kuwait provides an important case study for evaluating the interaction between cardiometabolic mortality and health system development within a high-income Middle Eastern setting. Between 2010 and 2022, the country experienced sustained growth in healthcare financing and cardiovascular service capacity while also facing pandemic-related disruption. Whether cardiometabolic mortality trends reflect underlying pre-pandemic escalation, acute pandemic-era inflection, or heterogeneous trajectories across disease subcategories remains insufficiently examined at the national level. Understanding these dynamics is essential for strengthening NCD prevention and control strategies in rapidly developing health systems.

The present study conducts a national ecological time-series analysis of cardiometabolic mortality in Kuwait from 2010 to 2022. Specifically, it examines trends in all-cause mortality, diseases of the circulatory system, ischaemic heart disease (IHD), cerebrovascular disease, hypertensive disease, and diabetes mellitus, alongside indicators of health system expansion including MOH expenditure and CCU capacity. By incorporating pre-COVID restricted analyses, this study aims to clarify whether observed cardiometabolic mortality patterns reflect sustained pre-pandemic escalation, pandemic-associated inflection, or heterogeneous trajectories across disease subcategories in the context of sustained health system expansion.

## 2. Materials and Methods

### 2.1. Study Design and Setting

This study employed a retrospective national ecological time-series design to examine trends in cardiometabolic mortality and health system expansion in Kuwait from 2010 to 2022. Kuwait is a high-income country with a predominantly publicly funded health system, where the Ministry of Health (MOH) is the principal provider of inpatient, outpatient, emergency, and preventive health services. This design is appropriate for evaluating longitudinal health system and mortality patterns using routinely collected administrative data and is consistent with established guidance for population-level time-series analyses in public health research [17,18,19].

### 2.2. Data Sources

Data were obtained from official MOH annual statistical reports and administrative datasets covering calendar years 2010–2022, with fiscal-year data used where applicable for health financing indicators [20]. The following data domains were included: Kuwait operates on an April–March fiscal year cycle. Health financing data (MOH budget and expenditure) were assigned to the ending calendar year of the corresponding fiscal period (e.g., FY 2021/2022 was treated as calendar year 2022), consistent with MOH report labelling. This introduces a minor three-month temporal offset that does not materially affect long-term trend analysis.

1. Mortality data:
  Deaths by nationality, sex, age group, and cause of death, classified according to the ICD-10 mortality tabulation list (103 causes), including all-cause mortality and major non-communicable disease categories.

2. Population denominators:
  Mid-year population estimates by nationality and sex, used for rate calculations.

3. Health service utilization:
  Annual outpatient and emergency visits, inpatient discharges, coronary care unit (CCU) discharges, cardiac surgery procedures, and cardiac catheterization procedures.

4. Health system capacity:
  Number of hospitals, total hospital beds, unit-specific beds (including CCU beds), and specialized cardiovascular units.

5. Health financing:
  Total MOH expenditure, per capita health expenditure, and the proportion of the national budget allocated to the MOH.

All data sources were aggregated at the national level and were de-identified.

### 2.3. Outcome Measures

#### 2.3.1. Mortality Outcomes

- All-cause mortality (annual number of deaths).
- Cause-specific mortality focusing on:
  – Diseases of the circulatory system;
  – Ischaemic heart disease;
  – Cerebrovascular diseases;
  – Hypertensive diseases;
  – Diabetes mellitus.

Cause-specific outcomes were defined using the ICD-10 mortality tabulation categories used in MOH annual statistical reports: diseases of the circulatory system (I00–I99); ischaemic heart disease (I20–I25); cerebrovascular disease (I60–I69); hypertensive disease (I10–I13); and diabetes mellitus (E10–E14).

#### 2.3.2. Health System Indicators

- Outpatient and emergency visit volumes.
- Inpatient discharges from cardiovascular-related units.
- Procedural activity by category.
- Bed capacity (total and unit-specific).
- Health expenditure indicators.

### 2.4. Rate Calculations

- Crude death rates (CDR) were calculated as

CDR = (Deaths ÷ Mid-year population) × 1000

- Cause-specific mortality rates were calculated as

Cause-specific rate = (Cause-specific deaths ÷ Mid-year population) × 100,000

Crude rather than age-standardized rates were used to describe population-level mortality burden in the context of demographic change, including population growth and aging. This approach is consistent with national descriptive epidemiology and aligns with the study objective of assessing overall mortality trends rather than age-specific risk [3,21]. As a pre-specified sensitivity analysis, age-standardized mortality rates (ASRs) were calculated for the primary cause-specific outcomes using the WHO World Standard Population (2000–2025) applied to cause-specific death counts by age group for all study years 2010–2022. No data imputation was performed; all 13 annual observations for each cause were extracted directly from the corresponding MOH annual statistical reports. Age-stratified mid-year population denominators were obtained from the same reports, which present national population estimates from the Public Authority of Civil Information (PACI) stratified by age, sex, and nationality. ASRs were computed as: ASR = Σ(age-specific rate × standard population weight)/Σ(standard population weights) × 100,000. Cause-specific mortality rates and ASRs are expressed per 100,000 population. The overall crude death rate is expressed per 1000 population, consistent with MOH reporting conventions. Results are presented in Supplementary Table S1. Age-band mortality rates per 100,000 for three broad age groups (<45, 45–64, ≥65 years) and nationality-stratified crude rates are provided in Supplementary Tables S2 and S3.

### 2.5. Temporal Aggregation Strategy

To reduce year-to-year variability and enhance interpretability, mortality and health system indicators were summarized using five-year block means:

- 2010–2014;
- 2015–2019;
- 2020–2022.

The final block (2020–2022) was treated as a shorter interval to distinguish late-period changes, including the COVID-19 era, without selecting groupings based on statistical significance.

Sensitivity analyses using three-year blocks were conducted descriptively and are presented in the Supplementary Materials to assess the robustness of observed patterns.

### 2.6. Statistical Analysis

#### 2.6.1. Descriptive Analysis

Descriptive statistics were used to summarize:

- Annual counts and crude rates;
- Block means across the three time periods;
- Absolute and percentage changes between 2010 and 2022.

#### 2.6.2. Trend Analysis

Long-term temporal trends were assessed using ordinary least squares (OLS) linear regression, with calendar year treated as a continuous independent variable:

*Y_t_* = *β*_0_ + *β*_1_⋅Year + *ε_t_*

where

- *β*_1_ represents the average annual change;
- Two-sided *p*-values were reported;
- 95% confidence intervals (CI) were calculated for each β coefficient;
- R^2^ (coefficient of determination) was reported to describe model fit and the proportion of variance in the outcome explained by calendar year.

OLS regression was selected due to the limited number of annual observations (*n* = 13), the study’s focus on estimating overall directional trends in population-level indicators, and the descriptive ecological design. This approach is consistent with established guidance for health services time-series analyses where the primary goal is trend estimation rather than forecasting or causal inference [17,18,19]. To assess residual independence, the Durbin–Watson (DW) statistic was computed for each primary OLS model (critical values: *n* = 13, k = 1, α = 0.05: d_l_ = 1.08, d_u_ = 1.37). DW statistics, adjusted R^2^ values, and 95% confidence intervals for all slope estimates are reported in the regression tables. Residual plots were visually inspected and did not indicate marked non-linearity or gross heteroscedasticity; given the short annual series, these diagnostics were interpreted descriptively rather than formally. As a robustness check, Newey–West heteroscedasticity-and-autocorrelation consistent (HAC) standard errors with one lag were computed for three core outcomes: crude death rate, circulatory disease mortality rate, and diabetes mortality rate. Significance conclusions were unchanged under HAC correction for all three outcomes (Supplementary Table S4).

#### 2.6.3. Pre-COVID Restricted Models

To distinguish secular trends from pandemic-era deviations, secondary OLS regression models were estimated for restricted time windows. For mortality indicators, models were restricted to 2010–2019 (*n* = 10 observations). For cardiac catheterization, models were restricted to 2012–2019 (*n* = 8 observations) to reflect data availability. For CCU indicators, models covering 2014–2022 were estimated to correspond with the period of available CCU-specific data.

Comparing full-series and pre-COVID β coefficients allowed assessment of whether observed trends: (1) predated 2020, (2) were amplified during the 2020–2022 pandemic period, or (3) were primarily driven by pandemic-era inflection. This approach follows principles of segmented time-series interpretation without formally modeling a step-change intervention parameter [17,18]. To formally characterise the structural change at 2020, an interrupted time-series (ITS) step-change model was estimated for all primary outcomes: Yₜ = β_0_ + β_1_·Year + β_2_·I (Year ≥ 2020) + εₜ. Results are reported in Supplementary Table S5.

#### 2.6.4. Capacity–Utilization Structural Model

To examine alignment between infrastructure expansion and service delivery, a supplementary OLS regression model was specified with CCU bed count as the independent variable and annual CCU discharges as the dependent variable:

_CCU Discharges_t = _α_0 + _α_1_(CCU Beds_t_)_ + _ε_t

This model evaluated whether increases in CCU bed capacity were structurally associated with increases in inpatient cardiovascular utilization. In this model, CCU bed count served as the independent variable rather than calendar year. The α_1_ coefficient represents the estimated change in annual CCU discharges per additional CCU bed. The β coefficient, 95% CI, two-sided *p*-value, and R^2^ were reported.

#### 2.6.5. Log-Linear Financing Model

Given evidence of exponential growth in MOH expenditure, a log-transformed regression model was estimated:

_log(Expenditure_t_)_ = _γ_0 + _γ_1_(Year)_ + _ε_t

The exponentiated γ_1_ ^coefficient (eγ1)^ represents the approximate annual compound growth factor in expenditure. This model was used to characterize the rate of health financing expansion and complements the linear OLS model for interpretive completeness.

#### 2.6.6. Assumption Checks

- Time-series plots were visually inspected to assess linearity.
- Residual plots were examined for evidence of gross heteroscedasticity.
- Autocorrelation was assessed descriptively; given the exploratory, population-level nature of the analysis and the study’s descriptive aims, formal autoregressive modeling was not pursued.

#### 2.6.7. Statistical Thresholds

Statistical significance was defined as *p* < 0.05 (two-sided). However, given the descriptive ecological design and limited annual observations, results were interpreted with emphasis on the direction, magnitude, and consistency of trends across domains, model fit (R^2^), and concordance between full-series and pre-COVID restricted models, rather than statistical significance alone.

All regression models were univariable, with calendar year or CCU bed count as the sole predictor. No covariate adjustment was performed, consistent with the descriptive, ecological design and the population-level nature of the outcome measures.

#### 2.6.8. Software

Data management, rate calculations, descriptive analyses, and ordinary least squares regression analyses were conducted using Microsoft Excel (Microsoft 365).

### 2.7. Ethical Considerations

This study was approved by the Health Sciences Center Ethical Committee (HSCEC), Kuwait University (Approval No. VDR/EC–2025–113).

The study utilized aggregate, de-identified secondary data obtained from publicly available Ministry of Health statistical reports, including national mortality, health service utilization, health system capacity, and financing indicators covering the period 2010–2022. No individual-level data were accessed, and no direct contact with human participants occurred; therefore, informed consent was not required.

## 3. Results

The results are organized into four thematic domains: (1) all-cause mortality and crude death rates, (2) cause-specific cardiovascular and diabetes mortality, (3) health system capacity and utilization, and (4) health system financing. Temporal patterns are assessed across three five-year blocks—2010–2014 (baseline), 2015–2019 (pre-COVID expansion), and 2020–2022 (pandemic period)—with supplementary pre-COVID restricted regressions (2010–2019) reported alongside full-series models to isolate the independent contribution of the pandemic period.

### 3.1. All-Cause Mortality and Crude Death Rates

#### 3.1.1. Descriptive Temporal Patterns

Between 2010 and 2022, Kuwait experienced a marked and progressive increase in total mortality burden. Annual all-cause deaths rose from 5448 in 2010 to a study-period peak of 10,938 in 2021, before declining to 8041 in 2022, representing a 47.6% net increase over the full study period. The five-year block means demonstrate a clear stepwise escalation: 5735 deaths annually during 2010–2014, increasing to 6722 during 2015–2019, and reaching 9849 during 2020–2022. The 2020–2022 block mean exceeds the preceding block by more than 3100 deaths annually, indicating a substantial late-period mortality acceleration.

Crude death rates (CDR) followed a related but more nuanced trajectory. The CDR remained stable at approximately 1.52 deaths per 1000 population across both 2010–2014 and 2015–2019 (SD = 0.04 for both blocks), before rising to a block mean of 2.13 per 1000 during 2020–2022 (SD = 0.31), peaking at 2.4 per 1000 in 2021. By 2022, the CDR had partially recovered to 1.8 per 1000, representing a 20% net increase relative to 2010. The divergence between stable pre-2020 rates and elevated 2020–2022 rates indicates that population growth alone does not account for the rising mortality burden; rather, the data reflect both a demographic-driven increase in absolute deaths across the decade and a late-period intensification in mortality risk.

#### 3.1.2. Regression Trend Analysis

Full-series OLS regression (2010–2022) demonstrated a statistically significant upward trend in all-cause deaths (β = +373.5 deaths/year; 95% CI: 194.5–552.4; *p* = 0.001; R^2^ = 0.66), indicating that year explained approximately 66% of the variance in annual deaths. The pre-COVID restricted model (2010–2019) yielded an even stronger fit (β = +196.2 deaths/year; *p* < 0.001; R^2^ = 0.94), confirming that rising mortality counts predated 2020 and represent a genuine underlying trend, further amplified during the pandemic period.

In contrast, the CDR exhibited a significant upward trend only over the full 2010–2022 series (β = +0.049 per 1000/year; 95% CI: 0.011–0.088; *p* = 0.017; R^2^ = 0.42), while the pre-COVID restricted model showed no statistically significant trend (β = +0.002; *p* = 0.631; R^2^ = 0.03). This distinction indicates that the overall increase in crude mortality rate is primarily driven by late-period changes rather than a steady secular rise, consistent with the interpretation that the pre-2020 mortality increase largely reflects demographic expansion, while the post-2020 elevation represents additional population-level mortality risk (Table 1).

### 3.2. Cause-Specific Cardiovascular Mortality

#### 3.2.1. Diseases of the Circulatory System

Mortality rates for diseases of the circulatory system rose from 64.2 per 100,000 population in 2010 to 79.8 per 100,000 in 2022, a 24.3% relative increase. Block means show relative stability during 2010–2014 (mean: 61.6 per 100,000; SD = 3.2) and 2015–2019 (mean: 62.2 per 100,000; SD = 3.6), followed by a pronounced elevation during 2020–2022 (mean: 80.0 per 100,000; SD = 1.2)—an increase of nearly 18 deaths per 100,000 between the two most recent periods. Rates during 2020–2022 were consistently high at 81.3, 78.9, and 79.8 per 100,000, respectively, suggesting a durable shift rather than a transient spike.

Full-series regression confirmed a statistically significant upward trend (β = +1.56 per 100,000/year; 95% CI: 0.57–2.55; *p* = 0.005; R^2^ = 0.52). However, the pre-COVID restricted model (2010–2019) was not statistically significant (β = +0.17; *p* = 0.657; R^2^ = 0.03), indicating that the apparent long-term upward trend is driven primarily by the 2020–2022 period rather than gradual escalation over the preceding decade. This temporal pattern implies a structural mortality inflection beginning around 2020 (Table 2).

#### 3.2.2. Ischaemic Heart Disease: Death Counts and Mortality Rates

Ischaemic heart disease (IHD) death counts increased from 1188 in 2010 to a peak of 2271 in 2020, before declining to 1628 in 2022, representing a net increase of 440 deaths (+37.0%) across the study period. Block means rose consistently: 1226 deaths/year in 2010–2014 (SD = 93.8), 1556 deaths/year in 2015–2019 (SD = 269.9), and 1895 deaths/year in 2020–2022 (SD = 334.9). Importantly, IHD death counts represent one of the most stable long-term upward signals in the dataset, with the rise predating the pandemic period.

Full-series regression confirmed a significant increasing trend in IHD death counts (β = +68.4 deaths/year; 95% CI: 31.7–105.1; *p* = 0.002; R^2^ = 0.60). Critically, the pre-COVID restricted model remained statistically significant (β = +67.0 deaths/year; *p* = 0.007; R^2^ = 0.62), confirming that the rise in IHD burden began well before 2020 and continued thereafter. This distinguishes IHD from circulatory disease overall, where the mortality signal is predominantly pandemic era.

In contrast, IHD mortality rates per 100,000 population did not demonstrate a statistically significant linear trend across the full study period (β = +0.368/year; *p* = 0.392; R^2^ = 0.07). Rates fluctuated between 28.6 per 100,000 in 2016 and 47.1 per 100,000 in 2020, with period means of 32.43 in 2010–2014, 35.00 in 2015–2019, and 40.74 in 2020–2022. The divergence between significant count trends and non-significant rate trends underscores the importance of interpreting mortality burden using both absolute and population-adjusted measures: the increase in IHD deaths is real, but population growth has materially offset the rate-based escalation (Table 3).

#### 3.2.3. Cerebrovascular Disease Mortality

Cerebrovascular disease mortality demonstrated a declining pattern over the study period, falling from 14.66 per 100,000 in 2010 to 10.33 per 100,000 in 2022, an absolute reduction of 4.33 per 100,000 (−29.5%). The decline was most pronounced between the baseline period (2010–2014 mean: 11.64 per 100,000; SD = 1.81) and 2015–2019 (mean: 9.65 per 100,000; SD = 0.38). Rates stabilised during 2020–2022 (mean: 10.66 per 100,000; SD = 0.29), suggesting the declining trajectory levelled off in the pandemic period.

Despite this apparent downward pattern, the full-series linear regression did not reach conventional statistical significance (β = −0.191 per 100,000/year; 95% CI: −0.394–0.013; *p* = 0.064; R^2^ = 0.28). This borderline result suggests a meaningful directional decline with moderate inter-annual variability, likely reflecting genuine improvements in cerebrovascular care delivery alongside earlier high variability.

#### 3.2.4. Hypertensive Disease Mortality

Hypertensive disease mortality exhibited a statistically significant and sustained decline throughout the study period. The rate fell from 4.07 per 100,000 in 2010 to 2.35 per 100,000 in 2022, an absolute reduction of 1.72 per 100,000 (−42.3%). This declining trajectory was consistent across all time blocks: 3.69 per 100,000 in 2010–2014 (SD = 0.29), 3.14 per 100,000 in 2015–2019 (SD = 0.54), and 2.60 per 100,000 in 2020–2022 (SD = 0.30), notably continuing even during the pandemic years.

Regression analysis confirmed this significant declining trend (β = −0.113 per 100,000/year; 95% CI: −0.176–−0.050; *p* = 0.002; R^2^ = 0.59). The magnitude and consistency of this reduction across all three time blocks indicates a sustained and progressive decline in hypertensive disease mortality throughout the study period (Table 4).

### 3.3. Diabetes Mellitus Mortality

Among all cause-specific outcomes examined, diabetes mellitus demonstrated the most consistent, robust, and clinically significant upward trajectory. Death counts rose from 187 in 2010 to 410 in 2022, representing a 119.3% relative increase—the largest proportional change in any indicator in this analysis. Block means reflect dramatic stepwise escalation: 164.8 deaths/year during 2010–2014 (SD = 31.6), rising to 397.2 deaths/year in 2015–2019 (SD = 168.0), and 487.3 deaths/year in 2020–2022 (SD = 74.2), peaking at 558 deaths in 2020.

Diabetes mortality rates per 100,000 population showed a parallel and independent upward trajectory, rising from 5.24 per 100,000 in 2010 to 9.18 in 2022 (+75.2%), with a period peak of 11.96 per 100,000 in 2018. Block means increased from 4.38 per 100,000 in 2010–2014 (SD = 0.99), to 8.85 in 2015–2019 (SD = 3.53), to 10.48 in 2020–2022 (SD = 1.21). The fact that both death counts and mortality rates increased significantly independently of population growth distinguishes diabetes from IHD and indicates a genuine rise in diabetes-related mortality risk, rather than a demographic artefact.

Regression analyses confirmed highly significant upward trends in both mortality rate (β = +0.689 per 100,000/year; 95% CI: 0.326–1.052; *p* = 0.002; R^2^ = 0.61) and death counts (β = +36.5 deaths/year; *p* < 0.001; R^2^ = 0.67). The strong model fit (R^2^ > 0.60 for both indicators) and consistency across both metrics establish diabetes mortality as the strongest independent epidemiologic signal in the dataset. Unlike circulatory mortality, the increase in diabetes mortality is not confined to the pandemic period but reflects sustained escalation throughout the entire study period (Table 5).

### 3.4. Health System Capacity and Utilization

#### 3.4.1. Coronary Care Unit Bed Capacity

CCU bed capacity expanded substantially between 2014 and 2022, rising from 51 beds to 147 beds—an increase of 96 beds (+188.2%). Growth was stepwise across successive time blocks: a mean of 62.7 beds/year in 2014–2016 (SD = 10.7), increasing to 84.0 beds/year in 2017–2019 (SD = 20.8), and reaching 145.7 beds/year in 2020–2022 (SD = 2.3). The most pronounced single-year expansion occurred between 2019 and 2020, when bed count increased from 108 to 147.

Regression analysis confirmed a highly significant upward trend in CCU capacity (β = +13.4 beds/year; 95% CI: 8.97–17.83; *p* < 0.001; R^2^ = 0.88), with year alone explaining approximately 88% of the variance in annual bed availability.

#### 3.4.2. Coronary Care Unit Utilization

CCU discharges—used as a proxy for inpatient cardiovascular care utilization—increased from 4255 in 2014 to 9678 in 2022, an absolute rise of 5423 discharges (+127.5%). Utilization grew consistently across all periods: 5689 discharges/year in 2014–2016 (SD = 1297), 6925 in 2017–2019 (SD = 557), and 8792 in 2020–2022 (SD = 815), demonstrating sustained and accelerating demand for inpatient cardiac care.

Regression confirmed a highly significant positive time trend (β = +537 discharges/year; *p* < 0.001; R^2^ = 0.87). Supplementary regression of CCU discharges on CCU bed count demonstrated a strong structural association (β = +36.35 discharges per additional bed; 95% CI: 20.67–52.02; *p* < 0.001; R^2^ = 0.81), indicating that capacity expansion was closely mirrored by proportional increases in care delivery—system alignment rather than idle expansion (Table 6).

#### 3.4.3. Cardiac Catheterization Procedures

Cardiac catheterization data were available from 2012 onward. Procedure volumes rose from 1335 in 2012 to a pre-COVID peak of 2584 in 2018 before collapsing to just 181 in 2020—a 93.0% single-year decline reflecting severe pandemic-related service disruption. Volumes partially recovered to 1807 in 2021 and 1711 in 2022, remaining below pre-pandemic peaks. Period means were 1638 in 2012–2014 (SD = 364.3), 2190 in 2015–2019 (SD = 359.9), and 1233 in 2020–2022 (SD = 912.3).

The full-series regression (2012–2022) revealed no statistically significant overall trend (β = −18.4 procedures/year; *p* = 0.785; R^2^ = 0.009), a result driven by the extreme 2020 disruption. The pre-COVID restricted model (2012–2019) demonstrated a significant positive trend (β = +129.7 procedures/year; *p* = 0.043; R^2^ = 0.52), confirming meaningful pre-pandemic growth in catheterization capacity that was subsequently disrupted and has not fully recovered.

#### 3.4.4. Cardiac Surgery Procedures

Cardiac surgery volumes declined over the full study period from 1529 procedures in 2010 to 1007 in 2022, an absolute decrease of 522 procedures (−34.2%). Period means fell progressively: 1455 in 2010–2014 (SD = 437.8), 1250 in 2015–2019 (SD = 102.4), and 988 in 2020–2022 (SD = 90.0). The 2011 volume of 2191 represents an outlier likely reflecting post-backlog catchup, and subsequent years showed consistent reduction.

Full-series regression confirmed a statistically significant downward trend (β = −53.6 procedures/year; 95% CI: −95.1–−12.1; *p* = 0.016; R^2^ = 0.42). The pre-COVID restricted model did not reach statistical significance (β = −53.4; *p* = 0.134; R^2^ = 0.26), suggesting that while surgical volumes were declining pre-pandemic, the trend reached significance when pandemic-period reductions were incorporated. The concurrent decline in surgical volumes alongside pre-pandemic growth in catheterization may reflect an ongoing shift toward less invasive interventional management in Kuwait’s cardiac care pathway (Table 7).

#### 3.4.5. Cardiology Outpatient Visits

Cardiology outpatient visits showed substantial inter-annual variability throughout the study period, without a statistically significant overall linear trend. Annual visits ranged from 93,291 in 2020 to 191,970 in 2022. Period means remained broadly stable: 150,280 visits/year in 2010–2014 (SD = 31,115), 141,075 visits/year in 2015–2019 (SD = 18,582), and 141,127 visits/year in 2020–2022 (SD = 49,408), with the high SD in the final block reflecting the sharp pandemic-related contraction in 2020 followed by rapid recovery.

Neither the full-series regression (β = −655.7 visits/year; *p* = 0.778; R^2^ = 0.008) nor the pre-COVID model (β = −1815.1; *p* = 0.536; R^2^ = 0.050) reached statistical significance, indicating that cardiology outpatient demand remained broadly stable across the study period, interrupted only transiently by the pandemic. The recovery to 191,970 visits in 2022—the highest level in the dataset—suggests resilient and rebounding outpatient demand.

### 3.5. Health System Financing

Total Ministry of Health (MOH) expenditure increased substantially and consistently throughout the study period, rising from KD 791.6 million in 2010 to KD 2107.8 million in 2022, an absolute increase in KD 1316.2 million and a relative increase of 166.3%. Block means rose progressively: KD 999.7 million/year in 2010–2014 (SD = 150.1 million), KD 1749.1 million/year in 2015–2019 (SD = 155.4 million), and KD 2018.2 million/year in 2020–2022 (SD = 78.5 million), with the rate of absolute growth notably strongest during 2015–2019.

OLS regression demonstrated a very strong and highly significant linear increase in MOH expenditure (β = +KD 115.2 million/year; 95% CI: 96.5–133.9 million; *p* < 0.001; R^2^ = 0.94), with year alone explaining 94% of the annual variance in health expenditure. A complementary log-linear model confirmed exponential growth, yielding β = 0.0826 in log units/year (*p* < 0.001; R^2^ = 0.92), corresponding to approximately 8.3% compounding annual growth. The near-perfect fit of both linear and log-linear models confirms that MOH financing expanded in a sustained and structurally predictable manner across the full study period—independently of and without the abrupt inflection observed in mortality rates during 2020–2022 (Table 8).

### 3.6. Integrated Temporal Pattern Across Domains

Across mortality, financing, capacity, and utilization domains, three dominant temporal patterns characterize the study period:

First, a consistent long-term increase in total deaths and IHD death counts—evident from 2010 to 2019 and confirmed by pre-COVID regressions—reflects a sustained rise in cardiovascular mortality burden that predates and extends beyond the pandemic. Diabetes mortality represents the most pronounced manifestation of this pattern, with escalating rates and counts across all three time blocks.

Second, a pronounced late-period elevation (2020–2022) drives much of the observed increase in circulatory disease mortality rates and crude death rates. Pre-COVID restricted analyses demonstrate that these rate-based increases are not attributable to pre-pandemic trends, indicating a structural mortality inflection linked to the pandemic era.

Third, substantial expansion in MOH financing and CCU capacity occurred in parallel with increasing inpatient cardiovascular utilization across the full study period. The strong structural association between CCU bed growth and discharge volume (R^2^ = 0.81) indicates that increases in infrastructure capacity were closely mirrored by proportional increases in service utilization.

These three patterns coexist but do not uniformly align. Health financing expanded steadily and predictably throughout the period, while mortality changes were temporally heterogeneous—with diabetes rising continuously, IHD counts rising pre-pandemic, and circulatory mortality rates elevating acutely post-2020. Procedural and outpatient volumes showed mixed trajectories, reflecting the differential impact of the pandemic across service types. The concurrent expansion of health system investments and rising cardiometabolic mortality burden indicates parallel evolution across domains rather than a direct relationship between system inputs and population-level outcomes.

### 3.7. Summary of Regression Findings

Table 9 provides a consolidated summary of all regression models, reporting direction, coefficient, significance, and model fit across the four analytical domains.

### 3.8. Age-Standardized Mortality Rate Sensitivity Analysis

Age-standardized mortality rate (ASR) sensitivity analyses using the WHO World Standard Population (2000–2025) confirmed the directional findings of the crude-rate analyses for all primary outcomes (Supplementary Table S1). Age-standardized IHD rates fluctuated within a broadly comparable range (65.96–107.37 per 100,000), consistent with the interpretation that increases in IHD death counts were largely influenced by demographic expansion rather than increases in age-specific risk. Age-standardized diabetes rates showed an upward pattern from 24.2 per 100,000 in 2010, peaking at 51.1 in 2018 and remaining at 30.4 in 2022, supporting a persistent elevation in diabetes-related mortality not explained solely by demographic change. Age-standardized hypertensive disease rates declined from 19.1 to 9.5 per 100,000, and cerebrovascular rates declined from 61.5 to 26.4 per 100,000 over the study period, indicating substantial age-adjusted improvement in both conditions. The higher ASRs relative to crude rates are consistent with Kuwait’s unusually young total-population age structure, which is strongly influenced by the large non-Kuwaiti working-age population; age-standardization raises rate estimates toward the level of underlying age-specific risk in older age groups.

## 4. Discussion

### 4.1. Principal Findings

This national ecological time-series analysis provides an integrated assessment of cardiometabolic mortality trends alongside health system financing, capacity, and utilization in Kuwait between 2010 and 2022. Three principal findings emerge. First, total mortality burden increased substantially over the study period, with statistically significant upward trends in all-cause deaths and ischaemic heart disease (IHD) death counts, both of which predated the COVID-19 pandemic. Second, diabetes mortality demonstrated the most consistent and robust increase in any cause-specific outcome, rising significantly in both rates and counts across the full period. Third, substantial expansion in Ministry of Health (MOH) financing and cardiac care unit (CCU) capacity occurred in parallel with increased inpatient cardiovascular utilization. These findings suggest a parallel evolution of disease burden and health system response, with substantial heterogeneity across indicators.

### 4.2. All-Cause Mortality: Population Growth Versus Late-Period Acceleration

Crude death rates did not increase significantly during the pre-COVID period and only demonstrated statistical significance when 2020–2022 were included in the model. This divergence between counts and rates implies that much of the pre-2020 increase in absolute deaths reflects demographic expansion rather than rising mortality intensity. In contrast, the elevation in crude death rate during 2020–2022 indicates a genuine increase in population-level mortality risk—a pattern consistent with pandemic-era excess mortality documented across comparable settings.

Globally, excess mortality analyses during the COVID-19 pandemic have demonstrated that all-cause mortality may rise due to both direct infection and indirect mechanisms, including delayed care, disruption of chronic disease management, and health system strain [11,22]. Although our analysis does not attribute causality, the temporal inflection observed during 2020–2022 is consistent with international evidence of pandemic-era excess mortality and cause-of-death redistribution.

### 4.3. Circulatory Mortality: A Pandemic-Era Inflection

Mortality rates for diseases of the circulatory system exhibited a statistically significant upward trend across the full period; however, the pre-COVID regression was not significant. Block mean analysis showed relative stability from 2010 to 2019 (~62 per 100,000), followed by a marked increase during 2020–2022 (~80 per 100,000). This pattern indicates that the apparent long-term rise in circulatory mortality rates is largely driven by the 2020–2022 period rather than gradual escalation over the preceding decade.

International evidence has documented changes in cardiovascular mortality patterns during the pandemic, including reduced acute care presentations, delayed treatment, and shifts in coding practices [15,16]. The observed pattern in Kuwait is epidemiologically plausible within this broader global context, where systemic disruption during 2020–2022 contributed to excess cardiovascular mortality across multiple settings.

### 4.4. Ischaemic Heart Disease: Counts Versus Rates

IHD death counts increased significantly both before and after 2020, indicating a sustained increase in absolute IHD burden that predates the pandemic. However, IHD mortality rates did not demonstrate a statistically significant linear trend across 2010–2022, showing substantial inter-annual variability including a peak in 2020 followed by decline in 2021–2022. The divergence between significant count trends and non-significant rate trends underscores that population growth contributed materially to the rise in absolute deaths, even when underlying mortality risk remained unstable.

This count–rate divergence reflects a broader interpretive principle: in a population undergoing rapid demographic expansion, absolute mortality counts can rise substantially even when age-specific mortality risk is unchanged or declining [1,23]. The significant rise in IHD death counts therefore signals increasing absolute burden on the health system, while the variable rate pattern suggests a complex interplay between population structure, healthcare delivery, and acute systemic disruption.

### 4.5. Diabetes Mellitus: The Strongest Independent Signal

Among all cause-specific outcomes examined, diabetes demonstrated the most consistent and statistically robust increase, with both mortality rates and death counts rising significantly across the study period and strong model fit (R^2^ > 0.60 in both models). This finding is consistent with global epidemiologic evidence indicating a marked rise in diabetes-related mortality over recent decades.

Unlike circulatory mortality, diabetes mortality exhibited a progressive increase across all three time blocks rather than a late-period spike alone, suggesting a sustained escalation in diabetes-related mortality burden that cannot be explained solely by demographic expansion or pandemic-era disruption. The magnitude of the rate increase (+75%) represents the largest proportional change in any cause-specific indicator in this analysis.

The GBD 2021 Study reported substantial increases in diabetes burden worldwide, with projections suggesting continued growth through 2050 [24]. Pooled analyses of global population-representative studies confirm persistent upward trends in diabetes prevalence and treatment demand across all income settings [25]. Kuwait’s high and rising diabetes prevalence—documented at approximately 19–21% among Kuwaiti adults in cross-sectional studies [8]—situates the mortality signal identified here within a context of chronic, undertreated disease burden. The increasing diabetes mortality trend observed in Kuwait is also likely to contribute to the broader cardiovascular disease burden, given the established bidirectional relationship between diabetes and ischaemic heart disease.

### 4.6. Heterogeneity Within Cardiovascular Components

Hypertensive disease mortality declined significantly, and cerebrovascular mortality showed a downward trend that did not reach statistical significance. This internal heterogeneity strengthens the credibility of the findings: it demonstrates that the regression models are capable of detecting both increases and decreases depending on the specific outcome, arguing against systematic upward bias or coding artefact as explanations for the IHD and diabetes signals.

Divergent trajectories across cardiovascular subcategories may reflect heterogeneous effectiveness of secondary prevention programs, differential sensitivity to pandemic-era disruption, or variation in the temporal penetration of guideline-directed therapy. The declining hypertensive mortality trend in particular may reflect the cumulative effect of antihypertensive prescribing expansion documented across GCC health systems [10,11].

### 4.7. Health Financing and System Expansion

MOH expenditure increased dramatically over the study period, with a highly significant linear trend (R^2^ = 0.94) and an approximate annual compound growth rate of 8.3%, confirmed by both linear and log-linear models. World Bank data document that rising health expenditure per capita is characteristic of high-income settings undergoing health system expansion [9], and the near-linear trajectory in Kuwait suggests structural, long-term investment rather than episodic or reactive funding.

Critically, the financing trajectory does not exhibit the same abrupt inflection observed in circulatory mortality rates during 2020–2022. This divergence indicates that the late-period mortality elevation is unlikely to reflect a funding contraction, and instead points toward demand-side and epidemiological drivers—including pandemic-era care disruption and the sustained diabetes burden—as primary correlates of the mortality signal during this period.

### 4.8. Capacity Expansion and Utilization Alignment

CCU bed capacity increased significantly between 2014 and 2022, and CCU discharges increased in parallel. The supplementary regression of discharges on CCU bed count demonstrated a strong positive structural association (R^2^ = 0.81), indicating that infrastructure expansion was closely mirrored by inpatient utilization growth rather than yielding excess capacity.

From a systems perspective, this capacity–utilization alignment suggests that CCU expansion was functionally integrated into service delivery. However, observational time-series data cannot establish whether expansion drove utilization or whether rising cardiovascular demand prompted infrastructure investment—both directions of association are plausible, and established methodological guidance cautions against causal interpretation of temporal associations in ecological designs [17,18,19]. The temporal alignment between capacity growth and rising IHD death counts is nonetheless consistent with the hypothesis that increasing cardiovascular burden was associated with inpatient care demand.

### 4.9. Procedural Patterns: Structural Shifts in Cardiovascular Care

Cardiac surgery volumes declined significantly over the full period. In contrast, cardiac catheterization increased significantly during the pre-COVID period (2012–2019) but did not demonstrate a significant trend when the 2020–2022 period was included, suggesting that interventional cardiology services were expanding prior to 2020 but experienced disruption or plateau during the late period.

The divergence between declining surgical volumes and earlier growth in catheterization may reflect a structural shift in cardiovascular management toward percutaneous intervention—a pattern documented in international registry analyses of high-income health systems [26]. However, the data do not permit causal inference regarding procedural substitution, and pandemic-related service interruptions have also been documented in cardiovascular procedural volumes globally [15], which may account for part of the catheterization plateau observed after 2019.

### 4.10. Integrated Interpretation Across Domains

When mortality, financing, capacity, and utilization are examined jointly, a coherent system-level narrative emerges. Health financing expanded steadily and predictably throughout the period, while mortality changes were temporally heterogeneous: diabetes mortality rising continuously across all blocks, IHD death counts increasing pre-pandemic, and circulatory mortality rates elevating acutely post-2020. Procedural volumes showed differentiated trajectories, with surgery declining and catheterization showing pre-2020 growth. The coexistence of sustained disease burden escalation and expanding cardiovascular service capacity suggests that the MOH has been responding to growing NCD demand—yet the persistence of rising mortality across multiple indicators, and particularly the uninterrupted rise in diabetes mortality, indicates that system expansion has not been sufficient to offset the underlying epidemiological trajectory.

### 4.11. Public Health Implications

For Kuwait specifically, the sustained and accelerating diabetes mortality signal represents the most actionable finding. Diabetes mortality rose faster than any other cause-specific indicator, with a proportional rate increase of 75% over 13 years, yet the current analysis cannot determine whether this reflects worsening prevalence, inadequate treatment coverage, poor glycaemic control, increasing case fatality, or delayed diagnosis. This gap underscores the need for Kuwait’s MOH to strengthen diabetes surveillance systems, integrate HbA1c-based registries into national statistics, and evaluate the reach and effectiveness of existing diabetes management programs. The divergence between declining hypertensive mortality and rising IHD burden also merits attention as a potential indicator of heterogeneous success across NCD prevention efforts: gains in blood pressure control may not have translated into commensurate reductions in ischaemic events.

Beyond strengthening population-level surveillance and metabolic risk management, emerging cardiovascular imaging technologies may also contribute to earlier identification of cardiometabolic disease at the clinical level. Advanced echocardiographic modalities—particularly myocardial strain analysis, including global longitudinal strain derived from speckle-tracking echocardiography—have demonstrated the ability to detect subclinical myocardial dysfunction in individuals with cardiometabolic conditions even when conventional measures such as ejection fraction remain normal [27]. Meta-analytic evidence indicates that subclinical strain abnormalities are associated with increased risk of future cardiovascular events and mortality, supporting their potential role in earlier clinical risk stratification [28]. Three-dimensional echocardiography further enhances the characterization of early structural changes associated with cardiometabolic remodeling [29]. Artificial intelligence-assisted cardiac phenotyping may extend these capabilities by enabling automated identification of high-risk individuals, offering a scalable complement to population-level prevention programs. Together, these innovations could support earlier intervention for the high-risk cardiometabolic populations identified in this study as a priority for targeted action within Kuwait’s cardiometabolic care pathways.

At the regional level, the patterns identified here are consistent with GCC-wide evidence of rapid health system expansion occurring in parallel with an uncontrolled NCD burden [10,11,30]. The GCC countries share structural features that create shared vulnerabilities: high rates of obesity and physical inactivity, large expatriate populations with distinct risk profiles, and health systems historically oriented toward curative rather than preventive care. The finding that substantial MOH investment did not suppress cardiometabolic mortality growth echoes regional health system reviews calling for a reorientation toward primary prevention, community-based NCD management, and health workforce development [11,30]. This study also demonstrates the value of MOH administrative time-series data as a surveillance instrument in settings where population-based disease registries remain limited—a lesson applicable across the GCC region.

### 4.12. Strengths of the Study

This analysis benefits from:

- National coverage using official MOH administrative data across 13 consecutive years;
- Separate modeling of absolute death counts and population-adjusted mortality rates, enabling distinction between demographic and epidemiological drivers;
- Pre-COVID restricted regression models, allowing assessment of whether observed trends predated pandemic-era disruption;
- Integrated analysis across four domains (mortality, financing, capacity, utilization), providing a systems-level perspective not available from single-domain studies;
- Transparent reporting of non-significant findings and heterogeneous trends, reducing the risk of selective outcome reporting.

### 4.13. Limitations

Several limitations should be considered when interpreting these findings. First, primary analyses relied on crude mortality rates. Kuwait’s population includes a large and demographically distinct expatriate workforce whose age structure differs markedly from Kuwaiti nationals and fluctuates across years; crude rates therefore reflect a mixture of true mortality risk change and demographic compositional change. As a sensitivity analysis, age-standardized rates (ASRs) were calculated for the primary cause-specific outcomes using the WHO World Standard Population and are reported in Supplementary Table S1; ASR results confirmed the directional findings of the crude-rate analysis for all primary outcomes.

Second, the ecological design precludes individual-level inference. Population-level temporal associations between system indicators (financing, capacity) and mortality trends cannot be attributed to individuals or interpreted as evidence of causal mechanisms—a limitation inherent to all ecological time-series analyses [17,18,19].

Third, the 2020–2022 mortality inflection cannot be disaggregated into direct COVID-19 deaths versus indirect pandemic effects such as delayed care-seeking, diagnostic displacement, or changes in cause-of-death coding practices. MOH administrative mortality data do not permit cause-of-death validation against clinical records, and the magnitude of indirect mortality contributions during this period remains undetermined.

Fourth, the dataset contains no information on risk factor trajectories, comorbidity burden, glycaemic control, treatment coverage, or lifestyle indicators. The drivers of the diabetes mortality increase—whether worsening population-level prevalence, inadequate pharmacological treatment, poor disease management, or increasing case fatality—cannot be determined from administrative mortality data alone. This limits the actionability of the diabetes signal for specific intervention planning.

Fifth, all regression models were estimated using OLS with *n* = 13 annual observations, with Durbin–Watson (DW) diagnostics used to guide cautious interpretation. DW testing identified potential positive autocorrelation in two models: diabetes deaths (DW = 1.053) and the circulatory disease mortality rate full series (DW = 0.689); for these models, standard errors may be slightly underestimated, and *p*-values should be interpreted with appropriate caution. Most other primary models fell in the inconclusive or no-autocorrelation range. The directional finding for diabetes is corroborated across multiple complementary analyses. These results should therefore be interpreted as indicative of directional trends rather than precise inferential estimates [17,18,19]. Sixth, total-population analyses may mask heterogeneity between Kuwaiti nationals and non-Kuwaiti residents. The non-Kuwaiti workforce is predominantly composed of young adult males, introducing a healthy worker selection effect (IHD: 49.2 vs. 30.0 per 100,000; diabetes: 19.9 vs. 4.6 per 100,000 in 2022). Nationality-stratified longitudinal trend analysis represents an important direction for future research. Seventh, minor inter-annual variation in ICD-10 cause-of-death coding practices cannot be excluded, though the consistent use of the 103-cause tabulation framework across annual MOH reports supports reasonable comparability of estimates over time. Eighth, health financing data are reported on Kuwait’s April–March fiscal year and were assigned to the ending calendar year of each fiscal period; this introduces a minor three-month offset that is unlikely to affect conclusions from the long-term trend analysis.

## 5. Conclusions

This national ecological time-series analysis demonstrates that cardiometabolic mortality in Kuwait has not declined despite more than a decade of sustained health system expansion. Absolute mortality burden increased substantially between 2010 and 2022, driven in part by demographic growth. However, cause-specific patterns diverged markedly: ischaemic heart disease death counts rose prior to the COVID-19 pandemic; circulatory mortality rates increased sharply during 2020–2022; and diabetes mortality exhibited the most consistent and sustained escalation, with a 75% rise in crude rates progressing across all three time blocks independent of pandemic-era disruption. Ministry of Health financing, cardiac care unit capacity, and cardiovascular service utilization expanded steadily and in structural alignment throughout the same period.

Nevertheless, the coexistence of expanding capacity—particularly when concentrated in tertiary and acute care—with rising cardiometabolic mortality suggests that cardiometabolic mortality trends occurred alongside sustained health system expansion, consistent with the interpretation that upstream prevention and long-term metabolic risk management remain essential. These observational associations should not be interpreted causally. The findings point toward a widening gap between downstream service delivery and upstream metabolic risk control. The uninterrupted rise in diabetes mortality underscores the urgency of strengthening primary prevention, early detection, long-term glycaemic control, and community-based chronic disease management alongside continued tertiary cardiovascular investment.

For Kuwait and other Gulf Cooperation Council countries undergoing rapid health system modernization, integrated surveillance using administrative time-series data may serve as a valuable and immediately available tool for monitoring alignment between disease burden and system response. Future research incorporating age-standardized mortality rates, individual-level risk factor trajectories, and cause-of-death registry validation will be essential to clarify the drivers of the observed trends. Strengthening integrated, data-driven NCD policy—alongside a reorientation of investment toward upstream prevention and longitudinal metabolic risk management—must become a central priority if sustained reductions in cardiometabolic mortality are to be achieved.

## Figures and Tables

**Table 1:** All-Cause Mortality and Crude Death Rate: Trends and Regression Estimates (Kuwait, 2010–2022).

Indicator	2010	2010–2014 Mean	2015–2019 Mean	2020–2022 Mean	2022	% Change 2010–2022	β (Full Series)	95% CI	*p*-Value	R^2^	β (Pre-COVID)	*p* (Pre)
All-cause deaths (*n*)	5448	5735	6722	9849	8041	+47.6%	+373.5	194.5–552.4	0.001	0.66	+196.2	<0.001
Crude death rate (/1000)	1.5	1.52	1.52	2.13	1.8	+20.0%	+0.049	0.011–0.088	0.017	0.42	+0.002	0.631

Note. Pre-COVID period = 2010–2019 (*n* = 10). β = regression coefficient (OLS). All-cause deaths: pre-COVID β significant, confirming pre-pandemic trend. CDR: pre-COVID β non-significant, confirming late-period (2020–2022) dominance.

**Table 2:** Circulatory System Mortality Rate (/100,000): Trends and Regression Estimates (Kuwait, 2010–2022).

2010	2010–2014 Mean	2015–2019 Mean	2020–2022 Mean	2022	% Change	β (Full)	95% CI	*p*	R^2^	β (Pre-COVID)	*p* (Pre)
64.2	61.6	62.2	80.0	79.8	+24.3%	+1.56	0.57–2.55	0.005	0.52	+0.17	0.657

Note. Pre-COVID regression (2010–2019) is not significant (*p* = 0.657), confirming that the overall upward trend is largely attributable to the 2020–2022 pandemic period.

**Table 3:** Ischaemic Heart Disease—Death Counts and Mortality Rate (/100,000) (Kuwait, 2010–2022).

Indicator	2010	2010–2014 Mean	2015–2019 Mean	2020–2022 Mean	2022	% Change	β (Full)	95% CI	*p*	R^2^	β (Pre-COVID)	*p* (Pre)
Death counts (*n*)	1188	1226	1556	1895	1628	+37.0%	+68.4	31.7–105.1	0.002	0.60	+67.0	0.007
Mortality rate (/100,000)	33.31	32.43	35.00	40.74	36.47	+9.5% *	+0.368	—	0.392	0.07	—	—

Note. * Net rate change 2010–2022 is positive (+9.5%). The 2010 baseline rate (33.31 per 100,000) is lower than 2022 (36.47 per 100,000) when using the PACI mid-year population denominator consistently across all causes. IHD mortality rate regression: non-significant across full series. Pre-COVID regression for death counts significant, confirming pre-pandemic origin of trend.

**Table 4:** Cerebrovascular and Hypertensive Disease Mortality Rates (/100,000) (Kuwait, 2010–2022).

Outcome	2010	2010–2014 Mean	2015–2019 Mean	2020–2022 Mean	2022	% Change	β (Full)	95% CI	*p*	R^2^
Cerebrovascular disease	14.66	11.64	9.65	10.66	10.33	−29.5%	−0.191	−0.394–0.013	0.064	0.28
Hypertensive disease	4.07	3.69	3.14	2.60	2.35	−42.3%	−0.113	−0.176–−0.050	0.002	0.59

Note. Cerebrovascular regression: borderline non-significant (*p* = 0.064), indicating directional decline. Hypertensive disease regression: statistically significant downward trend (*p* = 0.002). Together, these divergent patterns demonstrate internal heterogeneity within cardiovascular subcategories.

**Table 5:** Diabetes Mellitus Mortality—Death Counts and Rates (/100,000) (Kuwait, 2010–2022).

Indicator	2010	2010–2014 Mean	2015–2019 Mean	2020–2022 Mean	2022	% Change	β (Full)	95% CI	*p*	R^2^
Death counts (*n*)	187	164.8	397.2	487.3	410	+119.3%	+36.5	19.6–53.4	<0.001	0.67
Mortality rate (/100,000)	5.24	4.38	8.85	10.48	9.18	+75.2%	+0.689	0.326–1.052	0.002	0.61

Note. Both death counts and mortality rate regression models significant, confirming that diabetes mortality increased independently of population growth. The 119.3% increase in death counts represents the largest proportional change in any cause-specific indicator in this analysis.

**Table 6:** CCU Capacity and Utilization: Annual Trends and Regression Estimates (Kuwait, 2014–2022).

Indicator	β	95% CI	*p*-Value	R^2^	Observations
CCU beds (per year)	+13.4 beds/year	8.97–17.83	<0.001	0.88	*n* = 9
CCU discharges (per year)	+537 discharges/year	—	<0.001	0.87	*n* = 9
CCU discharges vs. CCU beds	+36.35 per bed	20.67–52.02	<0.001	0.81	*n* = 9

Note. CCU data available from 2014 onward (*n* = 9). Period means: 2014–2016 (beds: 62.7; discharges: 5689); 2017–2019 (beds: 84.0; discharges: 6925); 2020–2022 (beds: 145.7; discharges: 8792).

**Table 7:** Procedural Utilization: Cardiac Catheterization and Surgery (Kuwait, 2010/2012–2022).

Procedure	Period	2010/2012–2014 Mean	2015–2019 Mean	2020–2022 Mean	% Change	β (Full)	*p* (Full)	R^2^ (Full)	β (Pre-COVID)	*p* (Pre)
Cardiac catheterization	2012–2022	1638	2190	1233	+28.2% *	−18.4	0.785	0.009	+129.7	0.043
Cardiac surgery	2010–2022	1455	1250	988	−34.2%	−53.6	0.016	0.42	−53.4	0.134

Note. * Catheterization % change calculated from 2012 to 2022. Pre-COVID catheterization regression (2012–2019) significant (*p* = 0.043), confirming pre-pandemic growth disrupted by COVID-19. Cardiac surgery full-series regression significant (*p* = 0.016).

**Table 8:** Ministry of Health Expenditure Trends (Kuwait, 2010–2022).

Indicator	2010 Value	2010–2014 Mean	2015–2019 Mean	2020–2022 Mean	2022 Value	% Change 2010–2022	β	95% CI	*p*	R^2^
Total expenditure (KD million)	791.6 M	999.7 M	1749.1 M	2018.2 M	2107.8 M	+166.3%	+115.2 M/year	96.5–133.9 M	<0.001	0.94
Log expenditure (log-linear model)	—	—	—	—	—	~8.3%/year	β = 0.083	0.066–0.099	<0.001	0.92

Note. Both linear and log-linear models confirm sustained exponential expenditure growth across the full period. The financing trajectory does not exhibit the abrupt 2020 inflection observed in mortality indicators, indicating structural long-term investment.

**Table 9:** Consolidated Regression Summary—All Indicators (Kuwait, 2010–2022).

Domain	Outcome	Period	β (per Year)	*p*-Value	R^2^	Trend
Mortality	All-cause deaths (*n*)	2010–2022	+373.5	0.001	0.66	↑ Significant
Mortality	All-cause deaths (*n*)	2010–2019	+196.2	<0.001	0.94	↑ Significant
Mortality	Crude death rate (/1000)	2010–2022	+0.049	0.017	0.42	↑ Significant
Mortality	Crude death rate (/1000)	2010–2019	+0.002	0.631	0.03	NS
CVD	Circulatory rate (/100 k)	2010–2022	+1.56	0.005	0.52	↑ Significant
CVD	Circulatory rate (/100 k)	2010–2019	+0.17	0.657	0.03	NS
CVD	IHD deaths (*n*)	2010–2022	+68.4	0.002	0.60	↑ Significant
CVD	IHD deaths (*n*)	2010–2019	+67.0	0.007	0.62	↑ Significant
CVD	IHD mortality rate (/100 k)	2010–2022	+0.368	0.392	0.07	NS
CVD	Cerebrovascular rate (/100 k)	2010–2022	−0.191	0.064	0.28	↓ Borderline
CVD	Hypertensive rate (/100 k)	2010–2022	−0.113	0.002	0.59	↓ Significant
Diabetes	Diabetes deaths (*n*)	2010–2022	+36.5	<0.001	0.67	↑ Significant
Diabetes	Diabetes rate (/100 k)	2010–2022	+0.689	0.002	0.61	↑ Significant
Capacity	CCU beds	2014–2022	+13.4 beds	<0.001	0.88	↑ Significant
Utilization	CCU discharges	2014–2022	+537	<0.001	0.87	↑ Significant
Utilization	CCU discharges vs. beds	2014–2022	+36.35/bed	<0.001	0.81	↑ Significant
Utilization	Cardiac catheterization	2012–2022	−18.4	0.785	0.01	NS
Utilization	Cardiac catheterization	2012–2019	+129.7	0.043	0.52	↑ Significant
Utilization	Cardiac surgery	2010–2022	−53.6	0.016	0.42	↓ Significant
Utilization	Cardiology outpatient visits	2010–2022	−655.7	0.778	0.01	NS
Financing	MOH expenditure (KD)	2010–2022	+115.2 M/year	<0.001	0.94	↑ Significant
Financing	Log MOH expenditure	2010–2022	+0.083	<0.001	0.92	↑ Significant

Note. NS = not statistically significant (*p* ≥ 0.05). ↑ = significant upward trend; ↓ = significant downward trend; ↓ Borderline = *p* = 0.064. All regressions are OLS with calendar year as predictor unless noted. CCU discharges vs. beds: predictor is CCU bed count, not year. Pre-COVID analyses restricted to 2010–2019 (*n* = 10) or 2012–2019 (*n* = 8) for catheterization.

## Data Availability

The data analyzed in this study were obtained from publicly available Ministry of Health (MOH) annual statistical reports covering the period 2010–2022. These reports are accessible through the official MOH website: https://www.moh.gov.kw/en/NCHI/Pages/AnnualReports.aspx (accessed on 28 May 2025) (MOH Annual Health Statistical Reports, 47th to 59th editions, 2010–2022). No new individual-level datasets were generated. Derived analytical datasets used for regression analyses are available from the corresponding author upon reasonable request, subject to applicable institutional data-sharing policies.

## Supplementary Materials

The following supporting information can be downloaded at https://www.mdpi.com/article/10.3390/jcm15072697/s1. Table S1: Age-Standardized Mortality Rates, Kuwait 2010–2022. Table S2: Age-Band Mortality Rates per 100,000 (IHD and Diabetes Mellitus), Kuwait 2010–2022. Table S3: Nationality-Stratified Crude Mortality Rates (IHD and Diabetes Mellitus), Kuwait 2010–2022. Table S4: Newey–West HAC Robustness Check — Core Outcomes. Table S5: Interrupted Time-Series Step-Change Model, Kuwait 2010–2022. Table S6: ICD-10 Mortality Tabulation Categories for Primary Outcomes.

## Abbreviations

The following abbreviations are used in this manuscript:

CCU	Coronary Care Unit
CDR	Crude Death Rate
CI	Confidence Interval
COVID-19	Coronavirus Disease 2019
CVD	Cardiovascular Disease
GBD	Global Burden of Disease
GCC	Gulf Cooperation Council
HbA1c	Glycated Hemoglobin
ICD-10	International Classification of Diseases, 10th Revision
IDF	International Diabetes Federation
IHD	Ischaemic Heart Disease
KD	Kuwaiti Dinar
MOH	Ministry of Health
NCD	Non-Communicable Disease
OLS	Ordinary Least Squares
R^2^	Coefficient of Determination
SD	Standard Deviation
WHO	World Health Organization
