# Supplementary material for: Cardiometabolic Mortality and Health System Expansion in Kuwait (2010–2022): A National Time-Series Analysis
Source: J Clin Med. 2026 Apr 2;15(7):2697. doi: 10.3390/jcm15072697 (PMC13072817; doi:10.3390/jcm15072697)
Supplement: Supplementary file 1 [file jcm-15-02697-s001.zip › jcm-4200180-supplementary.pdf]

Supplementary Material

Cardiometabolic Mortality and Health System Expansion in Kuwait (2010–2022):

A National Time-Series Analysis

Journal of Clinical Medicine • Manuscript ID: jcm-4200180

Contents

**Table S1**	Age-Standardized Mortality Rates (WHO World Standard Population), Kuwait 2010–2022
**Table S2**	Age-Band Mortality Rates per 100,000 (IHD and Diabetes Mellitus), Kuwait 2010–2022
**Table S3**	Nationality-Stratified Crude Mortality Rates (IHD and Diabetes Mellitus), Kuwait 2010–2022
**Table S4**	Newey–West HAC Robustness Check — Core Outcomes
**Table S5**	Interrupted Time-Series Step-Change Model Results, Kuwait 2010–2022
**Table S6**	ICD-10 Mortality Tabulation Categories for Primary Outcomes (Appendix A)

Supplementary Table S1. Age-Standardized Mortality Rates, Kuwait 2010–2022

WHO World Standard Population (2000–2025); rates per 100,000 population.

Year	IHD		Diabetes mellitus		Hypertensive disease		Cerebrovascular disease	
	ASR	Crude	ASR	Crude	ASR	Crude	ASR	Crude
2010	93.29	33.31	24.15	5.24	19.09	4.07	61.50	14.66
2011	95.74	34.55	20.13	4.71	17.15	3.69	42.63	10.99
2012	95.15	33.99	22.40	5.28	16.67	3.86	47.41	11.90
2013	72.49	27.80	13.84	3.55	13.80	3.34	38.94	10.43
2014	76.31	32.50	13.00	3.14	14.95	3.49	36.18	10.20
2015	84.32	35.81	13.99	3.18	10.07	2.49	31.47	9.25
2016	65.96	28.64	40.23	9.33	18.26	3.76	35.31	10.28
2017	72.49	32.36	32.56	8.23	14.57	3.43	32.16	9.60
2018	79.47	35.96	51.10	11.96	11.16	2.67	31.40	9.60
2019	100.31	42.23	44.91	11.57	14.70	3.35	28.85	9.52
2020	107.37	47.14	39.44	11.58	10.92	2.93	28.05	10.82
2021	91.52	38.62	37.95	10.67	8.65	2.51	27.54	10.83
2022	90.80	36.47	30.42	9.18	9.54	2.35	26.38	10.33

ASR = age-standardized mortality rate. Formula: ASR = [Σ(age-specific rate × W_i) / Σ(W_i)] × 100,000, where W_i = WHO World Standard Population weights (WHO/GPE Discussion Paper No. 31, 2001; 18 age bands, 0–4 merged, summing to 1,000,000). Cause-specific death counts used as ASR numerators were extracted from MOH annual statistical reports (ICD-10 Mortality Tabulation List, 103 causes); unstated-age deaths were excluded from age-specific rate calculations. Crude rates shown alongside ASRs use total mid-year population denominators from MOH annual statistical reports. Note: IHD crude rates have been corrected to use the mid-year population denominator consistent with all other cause-specific rates. Cause-specific crude rates are per 100,000 population; the overall crude death rate used in regression analyses is expressed per 1,000 population (MOH convention). IHD = ischaemic heart disease. All 13 annual observations are derived directly from MOH annual statistical reports; no imputation was performed.

Interpretation: Age-standardized IHD rates fluctuated within a broadly comparable range (65.96–107.37 per 100,000), consistent with demographic expansion rather than increased age-specific risk. Age-standardized diabetes rates showed an upward pattern from 24.2 per 100,000 in 2010, peaking at 51.1 in 2018, supporting a persistent elevation not explained solely by demographic change. Age-standardized hypertensive and cerebrovascular rates both declined over the study period, indicating age-adjusted improvement in these conditions. Directional conclusions of the crude-rate analysis are preserved under age-standardization for all primary outcomes.

Supplementary Table S2. Age-Band Mortality Rates per 100,000, Kuwait 2010–2022

Ischaemic heart disease (IHD) and diabetes mellitus. Stratified by age group (<45, 45–64, ≥65 years) and nationality.

Year	Total Population			Kuwaiti Nationals			Non-Kuwaiti Residents		
**Year**	**<45**	**45–64**	**≥65**	**<45**	**45–64**	**≥65**	**<45**	**45–64**	**≥65**
**Panel A: Ischaemic Heart Disease (IHD) — Mortality Rates per 100,000**									
**2010**	7.4	95.5	646.6	4.5	100.6	702.7	8.8	93.8	570.4
**2011**	8.7	92.3	662.6	6.7	99.8	755.2	9.7	89.8	537.2
**2012**	6.9	90.9	694.2	4.2	97.4	798.9	8.2	88.8	554.6
**2013**	5.8	75.1	497.6	3.3	68.8	578.6	7.0	77.1	389.7
**2014**	8.4	90.1	494.4	4.9	91.3	557.9	10.0	89.7	410.7
**2015**	8.9	93.7	555.6	5.7	106.8	591.0	10.4	89.6	510.3
**2016**	7.4	73.8	443.1	4.8	78.6	457.2	8.6	72.3	425.5
**2017**	8.9	82.2	475.0	5.9	83.2	522.5	10.3	81.9	415.7
**2018**	9.8	89.0	517.7	7.9	95.0	530.8	10.7	87.1	501.5
**2019**	9.3	99.9	688.0	5.7	96.9	707.1	11.0	100.8	664.0
**2020**	6.9	116.4	726.1	4.9	111.6	752.9	7.8	117.8	694.2
**2021**	4.5	87.3	637.7	3.3	90.3	751.2	5.1	86.4	499.0
**2022**	4.4	74.0	654.4	3.4	89.1	699.3	5.0	69.2	587.4
**Panel B: Diabetes Mellitus — Mortality Rates per 100,000**									
**2010**	0.4	7.5	197.7	0.6	16.2	250.6	0.4	4.6	125.9
**2011**	0.2	6.6	182.6	0.3	17.4	250.9	0.1	3.0	90.1
**2012**	0.1	8.6	190.3	0.2	21.2	272.0	0.1	4.5	81.6
**2013**	0.1	5.5	122.1	0.2	15.1	151.1	0.0	2.3	83.5
**2014**	0.1	4.0	110.7	0.0	8.4	152.8	0.1	2.5	55.3
**2015**	0.1	4.7	106.7	0.1	12.6	148.3	0.0	2.2	53.4
**2016**	0.3	10.0	337.3	0.6	25.8	465.2	0.2	5.1	178.1
**2017**	0.3	11.2	272.2	0.4	25.3	376.8	0.3	6.8	141.7
**2018**	0.4	13.8	403.9	0.8	29.3	534.4	0.2	9.0	244.2
**2019**	0.5	13.4	372.3	0.7	27.8	507.4	0.5	8.9	201.7
**2020**	0.5	13.3	329.9	0.7	32.1	454.2	0.5	7.9	182.5
**2021**	0.5	10.6	304.9	1.0	22.2	433.7	0.3	7.1	147.5
**2022**	0.4	9.8	254.5	0.7	26.3	308.0	0.3	4.5	174.7

Rates per 100,000 population. Age groups: <45 (ages 0–44); 45–64; ≥65. Deaths and population denominators from MOH annual statistical reports. Male and female deaths summed within each group. Nationality breakdown: Kuwaiti nationals vs. non-Kuwaiti residents. All 13 annual observations are derived directly from MOH reports; no imputation was performed.

Interpretation: Diabetes mortality is concentrated almost entirely in the ≥65 age group (197.7 per 100,000 in 2010, peaking at 403.9 per 100,000 in 2018 for the total population), with negligible rates in those aged <45. IHD mortality is distributed across working-age groups (45–64), with the ≥65 group contributing disproportionately. Kuwaiti nationals carry 1.2–1.65 times the IHD mortality rate of non-Kuwaiti residents, and 4–6 times the diabetes mortality rate, confirming concentration of chronic disease burden within the Kuwaiti subpopulation.

Supplementary Table S3. Nationality-Stratified Crude Mortality Rates, Kuwait 2010–2022

IHD and diabetes mellitus, Kuwaiti nationals vs. non-Kuwaiti residents.

Year	IHD (per 100,000)			Diabetes mellitus (per 100,000)			Δ vs. 2010 baseline (Kuwaiti rate)	
**Year**	**Kuwaiti**	**Non-Kw**	**Ratio**	**Kuwaiti**	**Non-Kw**	**Ratio**	**ΔIHD**	**ΔDM**
**2010**	38.7	29.4	1.32	10.6	2.5	4.24	—	—
**2011**	43.3	29.8	1.45	10.8	1.7	6.35	+4.6	+0.2
**2012**	43.1	28.9	1.49	12.1	1.8	6.72	+4.4	+1.5
**2013**	31.9	24.4	1.31	7.4	1.5	4.93	−6.8	−3.2
**2014**	36.2	29.7	1.22	6.6	1.3	5.08	−2.5	−4.0
**2015**	41.0	32.1	1.28	7.3	1.1	6.36	+2.3	−3.3
**2016**	32.2	26.7	1.21	21.7	3.5	6.20	−6.5	+11.1
**2017**	36.9	30.1	1.23	18.6	3.5	5.31	−1.8	+8.0
**2018**	41.5	33.3	1.25	26.5	5.5	4.82	+2.8	+15.9
**2019**	48.7	39.2	1.24	26.1	5.2	5.02	+10.0	+15.5
**2020**	55.2	43.5	1.27	26.2	5.1	5.14	+16.5	+15.6
**2021**	50.4	32.7	1.54	23.9	4.4	5.43	+11.7	+13.3
**2022**	49.2	29.8	1.65	19.0	4.1	4.63	+10.5	+8.4

Rates per 100,000 population. Ratio = Kuwaiti rate ÷ Non-Kuwaiti rate. Δ = absolute change in Kuwaiti rate from 2010 baseline (reference year). IHD = ischaemic heart disease; DM = diabetes mellitus. Population denominators from MOH annual statistical reports; all 13 observations directly extracted with no imputation. Non-Kuwaiti workforce predominantly composed of young adult males, introducing a healthy worker selection effect that depresses non-Kuwaiti mortality rates.

Interpretation: Kuwaiti nationals consistently carry a substantially higher cardiometabolic mortality burden than non-Kuwaiti residents. The Kuwaiti diabetes rate rose from 10.6 per 100,000 in 2010 to 26.1 in 2019, a ~141% increase, before declining modestly. The nationality ratio for diabetes (4–6× throughout the period) reflects both differential disease prevalence and the healthy worker demographic structure of the non-Kuwaiti workforce. Nationality-stratified longitudinal trend analysis is recommended as an important next step for future research.

Supplementary Table S4. Newey–West HAC Robustness Check — Core Outcomes

Heteroscedasticity-and-autocorrelation consistent (HAC) standard errors, lag = 1.

Outcome	n	β (slope)	SE (OLS)	SE (HAC)	p (OLS)	p (HAC)	Conclusion
**Crude death rate[1]**	13	+0.0495/yr	0.0177	0.0189	0.017	0.024	Unchanged
**Circulatory mortality rate**	13	+1.559/yr	0.451	0.489	0.005	0.009	Unchanged
**Diabetes mortality rate**	13	+0.689/yr	0.165	0.147	0.002	0.001	Unchanged

HAC = heteroscedasticity-and-autocorrelation consistent. SE(OLS) = conventional OLS standard error. SE(HAC) = Newey–West HAC standard error with one lag. p-values for OLS and HAC computed from their respective standard errors. Conclusion refers to whether statistical significance at $\alpha = 0.05$ is altered by HAC correction. [1] Crude death rate is expressed per 1,000 population per year, consistent with MOH reporting conventions; all other rates are per 100,000 population per year. Primary OLS regression results are retained in the main regression tables for consistency across the full set of outcomes.

Interpretation: Significance conclusions are unchanged under HAC correction for all three core outcomes. Crude death rate: p = 0.017 (OLS) vs. 0.024 (HAC). Circulatory mortality rate: p = 0.005 vs. 0.009. Diabetes mortality rate: p = 0.002 vs. 0.001. HAC standard errors are within ±15% of OLS standard errors, indicating that autocorrelation does not materially affect inferential precision for the primary trend estimates.

Supplementary Table S5. Interrupted Time-Series Step-Change Model, Kuwait 2010–2022

Model: $Y_t = \beta_0 + \beta_1 \cdot \text{Year} + \beta_2 \cdot I(\text{Year} \geq 2020) + \varepsilon_t$, where I(Year≥2020) is a binary indicator equal to 1 for years 2020–2022.

β_0 Outcome	n	β_1 (Year)	p (Year)	β_2 (Step ≥2020)	95% CI Step	p (Step)	R^2
**All-cause deaths**	13	+161.7/yr	0.088	+2,570 deaths	[880, 4,259]	0.007	0.841
**Crude death rate[1]**	13	−0.002/yr	0.881	+0.629/1,000	[0.32, 0.94]	0.001	0.811
**Circulatory disease rate**	13	+0.148/yr	0.665	+17.12/100k	[10.56, 23.68]	<0.001	0.891
IHD death counts	13	+57.8/yr	0.045	+128.5 deaths	[−372, 629]	0.580	0.617
IHD mortality rate	13	+0.026/yr	0.967	+4.15/100k	[−8.09, 16.40]	0.467	0.118
Diabetes mortality rate	13	+0.799/yr	0.010	−1.34/100k	[−6.28, 3.60]	0.560	0.627
Diabetes death counts	13	+42.1/yr	0.005	−67.2 deaths	[−296, 162]	0.528	0.686
Hypertensive disease rate	13	−0.099/yr	0.047	−0.18/100k	[−1.04, 0.69]	0.657	0.596
**Cerebrovascular rate**	13	−0.413/yr	0.003	+2.70/100k	[0.63, 4.78]	0.016	0.608

Model estimated by OLS; n = 13 for all outcomes. β_1 = annual secular trend coefficient. β_2 = step-change coefficient at 2020. Highlighted rows (shaded orange): statistically significant step-change coefficient ($p < 0.05$). 95% CI refers to the confidence interval for β_2. [1] Crude death rate per 1,000 population per year (MOH convention); all other rates per 100,000 per year.

Interpretation: Significant step changes at 2020 are observed for circulatory disease mortality rate (β_2 = +17.12/100k, $p < 0.001$), all-cause deaths (β_2 = +2,570, $p = 0.007$), crude death rate (β_2 = +0.629/1,000, $p = 0.001$), and cerebrovascular rate (β_2 = +2.70/100k, $p = 0.016$). Critically, the diabetes mortality step-change coefficient is non-significant (β_2 = −1.34, $p = 0.560$) while the secular trend remains significant (β_1 = +0.799/yr, $p = 0.010$), formally confirming that the diabetes increase reflects an independent secular escalation rather than pandemic-period disruption. IHD counts show a significant secular trend (β_1 = +57.8/yr, $p = 0.045$) with no significant step change.

Supplementary Table S6 (Appendix A). ICD-10 Mortality Tabulation Categories for Primary Outcomes

ICD-10 classifications used in Kuwait MOH Annual Health Statistical Reports.

Cause of Death / Outcome	ICD-10 Codes	Included Conditions and Sub-categories
Diseases of the circulatory system	**I00–I99**	All diseases of the circulatory system; encompasses ischaemic heart disease, cerebrovascular disease, hypertensive disease, rheumatic heart disease, and other cardiac conditions.
Ischaemic heart disease (IHD)	**I20–I25**	Angina pectoris (I20), acute myocardial infarction (I21), subsequent myocardial infarction (I22), complications of acute MI (I23), other acute IHD (I24), and chronic IHD (I25).
Hypertensive disease	**I10–I13**	Essential (primary) hypertension (I10), hypertensive heart disease (I11), hypertensive renal disease (I12), and hypertensive heart and renal disease (I13).
Cerebrovascular disease	**I60–I69**	Subarachnoid haemorrhage (I60), intracerebral haemorrhage (I61), other non-traumatic intracranial haemorrhage (I62), cerebral infarction (I63), stroke NOS (I64), and sequelae of cerebrovascular disease (I69).
Diabetes mellitus	**E10–E14**	Type 1 diabetes mellitus (E10), Type 2 diabetes mellitus (E11), malnutrition-related diabetes mellitus (E12), other specified diabetes mellitus (E13), and unspecified diabetes mellitus (E14).

Classifications follow the ICD-10 Mortality Tabulation List (103 causes) as applied in Kuwait MOH annual statistical reports. The same classification framework was used consistently across all 13 study years (2010–2022), as confirmed by table headers in each annual edition. This consistency supports reasonable comparability of cause-specific death counts across years; minor year-to-year coding variation cannot be excluded but is considered unlikely to materially affect trend interpretation. ICD-10 = International Classification of Diseases, 10th Revision.
